# Characterization and virulence clustering analysis of extraintestinal pathogenic *Escherichia coli* isolated from swine in China

**DOI:** 10.1186/s12917-017-0975-x

**Published:** 2017-04-08

**Authors:** Yinchu Zhu, Wenyang Dong, Jiale Ma, Lvfeng Yuan, Hassan M.A. Hejair, Zihao Pan, Guangjin Liu, Huochun Yao

**Affiliations:** 1 College of Veterinary Medicine, Nanjing Agricultural University, Nanjing, 210095 China; 2 Key Lab of Animal Bacteriology, Ministry of Agriculture, Nanjing Agricultural University, Nanjing, 210095 China

**Keywords:** Porcine extraintestinal pathogenic *Escherichia coli*, Genotype, Virulence, Multilocus sequence typing

## Abstract

**Background:**

Swine extraintestinal pathogenic *Escherichia coli* (ExPEC) is an important pathogen that leads to economic and welfare costs in the swine industry worldwide, and is occurring with increasing frequency in China. By far, various virulence factors have been recognized in ExPEC. Here, we investigated the virulence genotypes and clonal structure of collected strains to improve the knowledge of phylogenetic traits of porcine ExPECs in China.

**Results:**

We isolated 64 Chinese porcine ExPEC strains from 2013 to 14 in China. By multiplex PCR, the distribution of isolates belonging to phylogenetic groups B1, B2, A and D was 9.4%, 10.9%, 57.8% and 21.9%, respectively. Nineteen virulence-related genes were detected by PCR assay; *ompA*, *fimH*, *vat*, *traT* and *iutA* were highly prevalent. Virulence-related genes were remarkably more prevalent in group B2 than in groups A, B1 and D; notably, *usp*, *cnf1*, *hlyD*, *papA* and *ibeA* were only found in group B2 strains. Genotyping analysis was performed and four clusters of strains (named I to IV) were identified. Cluster IV contained all isolates from group B2 and Cluster IV isolates had the strongest pathogenicity in a mouse infection model. As phylogenetic group B2 and D ExPEC isolates are generally considered virulent, multilocus sequence typing (MLST) analysis was performed for these isolates to further investigate genetic relationships. Two novel sequence types, ST5170 and ST5171, were discovered. Among the nine clonal complexes identified among our group B2 and D isolates, CC12 and CC95 have been indicated to have high zoonotic pathogenicity. The distinction between group B2 and non-B2 isolates in virulence and genotype accorded with MLST analysis.

**Conclusion:**

This study reveals significant genetic diversity among ExPEC isolates and helps us to better understand their pathogenesis. Importantly, our data suggest group B2 (Cluster IV) strains have the highest risk of causing animal disease and illustrate the correlation between genotype and virulence.

**Electronic supplementary material:**

The online version of this article (doi:10.1186/s12917-017-0975-x) contains supplementary material, which is available to authorized users.

## Background

Extraintestinal pathogenic *Escherichia coli* (ExPEC) is a major pathogenic agent causing disease worldwide. It can lead to many kinds of extraintestinal diseases, including newborn meningitis, sepsis, and urinary tract disease [1]. It has been reported to cause death in animals and humans in Europe and North America [2, 3]. ExPEC can be distinguished from intestinal pathogenic *E. coli* and commensal *E. coli* by isolation site and genetic patterns [4]. Based on the isolation site, ExPEC can be classified as uropathogenic *E. coli* (UPEC), avian pathogenic *E. coli* (APEC), newborn meningitis *E. coli* (NMEC) or sepsis *E. coli* (SEPEC). The infectivity of ExPEC involves various extraintestinal virulence-associated factors in microbe-host interactions, rather than a simple mechanism [5]. Genome plasticity of ExPEC is the basis for rapid adaptation to the changing environment, which in turn increases the diversity of *E. coli* populations.

ExPEC is a major pathogen in the swine industry, responsible for considerable economic losses [6]. Alongside rapid development of industrial swine husbandry in China, an increasing trend of porcine ExPEC outbreaks has become an urgent problem [7]. Furthermore, the similarities in ExPEC isolates from human and animal infection cases suggest that there is cross-infection potential between different hosts, including humans, companion animals, pigs and birds [8]. I.e., ExPEC has high zoonotic potential, which makes porcine ExPEC a significant danger to public health.

*E. coli* consists of phylogenetic groups, namely A, B1, B2 and D. Most virulent ExPEC strains belong to group B2, followed by Group D. Generally, isolates assigned to groups A or B1 are less likely to cause disease in healthy animals [9]. The presence of virulence-related (VR) genes has a close connection with the virulence phenotype. Many genes have been identified as VR in ExPEC, and are involved in adhesion (P, S fimbriae, F1C fimbriae, Dr-binding adhesin and type I fimbriae), invasion (*ibeA*, invasive of brain endothelium), iron acquisition (siderophores), toxicity (hemolysin, vacuolating toxin, cytotoxic necrotizing factor) and surface protection (capsule) [10]. To identify combinations of VR genes associated with disease, the virulence genotyping method has previously been used in studies of UPEC [11]. However, such data is still limited for porcine ExPEC. In this study, we performed virulence genotyping, animal infection experiments and multilocus sequence typing (MLST) analysis, to provide insight into the associations between genotypes, phylogenetic groups and virulence phenotypes of ExPEC.

## Results

### Hemolytic activity tests

Of 64 tested ExPEC isolates, five produced β-hemolysin in 7% sheep blood agar, namely strains DCE-1, DCE-2, DCE-5, DCE-6, DCE-9.

### Prevalence of VFs

The prevalence of VR genes was diverse (Additional file 1). *iutA, vat, fimH, traT* and *ompA* appeared in >60% of strains (*ompA* was observed in all isolates). Among adhesion factors, *fimH* was the most prevalent (81.2%). Among siderophore factors, *iutA* (60.9%) was more frequently detected than *fyuA, ireA* and *iroN*. The prevalence of *cnf1, focG, ibeA, kpsMIII, afa, hlyD, sfaA, papA* was <10%, and *focG* was not detected in any isolate. Table 1 shows these results in detail.

**Table 1:** Distribution of virulence factors classified according to phylogenetic group

Virulence factors	NO. of ExPEC isolates(%)				
	Total (*n* = 64;%)	Group A (*n* = 37;57.8%)	GroupB1 (*n* = 6;9.4%)	GroupB2 (*n* = 7;10.9% )	Group D (*n* = 14;21.9%)
Siderophores					
*iutA*	39(60.1)	24(64.9)^a^	1(16.7)^b^	7(100)^b^	7(50)^b^
*iroN*	17(26.6)	4(10.9)^a^	3(50)^b^	6(85.7)^b^	4(28.6)^b^
*fyuA*	15(23.4)	5(13.5)^a^	0(0)^a^	7(100)^b^	3(21.4)^c^
*ireA*	7(10.9)	3(8.1)	0(0)	1(14.3)	3(21.4)
Adhesins					
*fimH*	52(81.3)	23(62.2)	6(100)	7(103)	11(78.6)
*papC*	14(21.9)	5(13.5)^a^	1(16.7)^ab^	5(71.4)^b^	3(21.4)^b^
*papA*	3(4.7)	0(0)^a^	0(0)^ab^	3(42.9)^b^	0(0)^b^
*sfaA*	1(1.6)	1(2.7)	0(0)	0(0)	0(0)
*hraA*	13(20.3)	4(10.9)^a^	0(0)^a^	5(71.4)^b^	4(28.6)^b^
*afa*	3(4.7)	3(8.1)	0(0)	0(0)	0(0)
Toxin					
*cnf1*	5(7.8)	0(0)^a^	0(0)^a^	5(71.4)^b^	0(0)^c^
*hlyD*	5(7.8)	0(0)^a^	0(0)^a^	5(71.4)^b^	0(0)^c^
*vat*	42(65.6)	23(62.2)	4(66.7)	7(100)	8(57.1)
Miscellaneous					
*ompA*	64(100)	37(100)	6(100)	7(100)	14(100)
*ups*	7(10.9)	0(0)^a^	0(0)^a^	7(100)^b^	0(0)^c^
*traT*	40(62.5)	30(81.1)^a^	4(66.7)^ab^	3(42.9)^b^	3(21.4)^b^
Invasion					
*ibeA*	2(3.1)	0(0)^a^	0(0)^ab^	2(28.6)^b^	0(0)^b^
Capsule					
*kpsMII*	10(15.7)	0(0)^a^	0(0)^a^	7(100)^b^	3(21.4)^c^
*kpsMIII*	1(1.6)	0(0)	0(0)	0(0)	1(7.1)

a,b,c *P* < 0.05

Phylogenetic group classification revealed that 9.4% (6/64) of the porcine ExPEC isolates belonged to group B1, 10.9% (7/64) to group B2, 57.8% (37/64) to group A and 21.9% (14/64) to group D. The phylogenetic group classification was associated with genetic patterns. The average number of VR genes in phylogenetic group B2 (12.1) was significantly higher than that in groups A (4.4), B1 (3.8), and D (4.3) (Fisher test, *P* < 0.05). *hlyD, ibeA, usp, cnf1, papA* were only observed in group B2 and *sfaA, afa* were only detected in group A. *ireA, papA, sfaA, afa, kpsMIII, ibeA* were rarely detected in any of the phylogenetic groups. Additional file 2: Table S1 shows statistical analysis of associations between virulence-associated factors.

### Cluster structure analysis

**Fig. 1:**
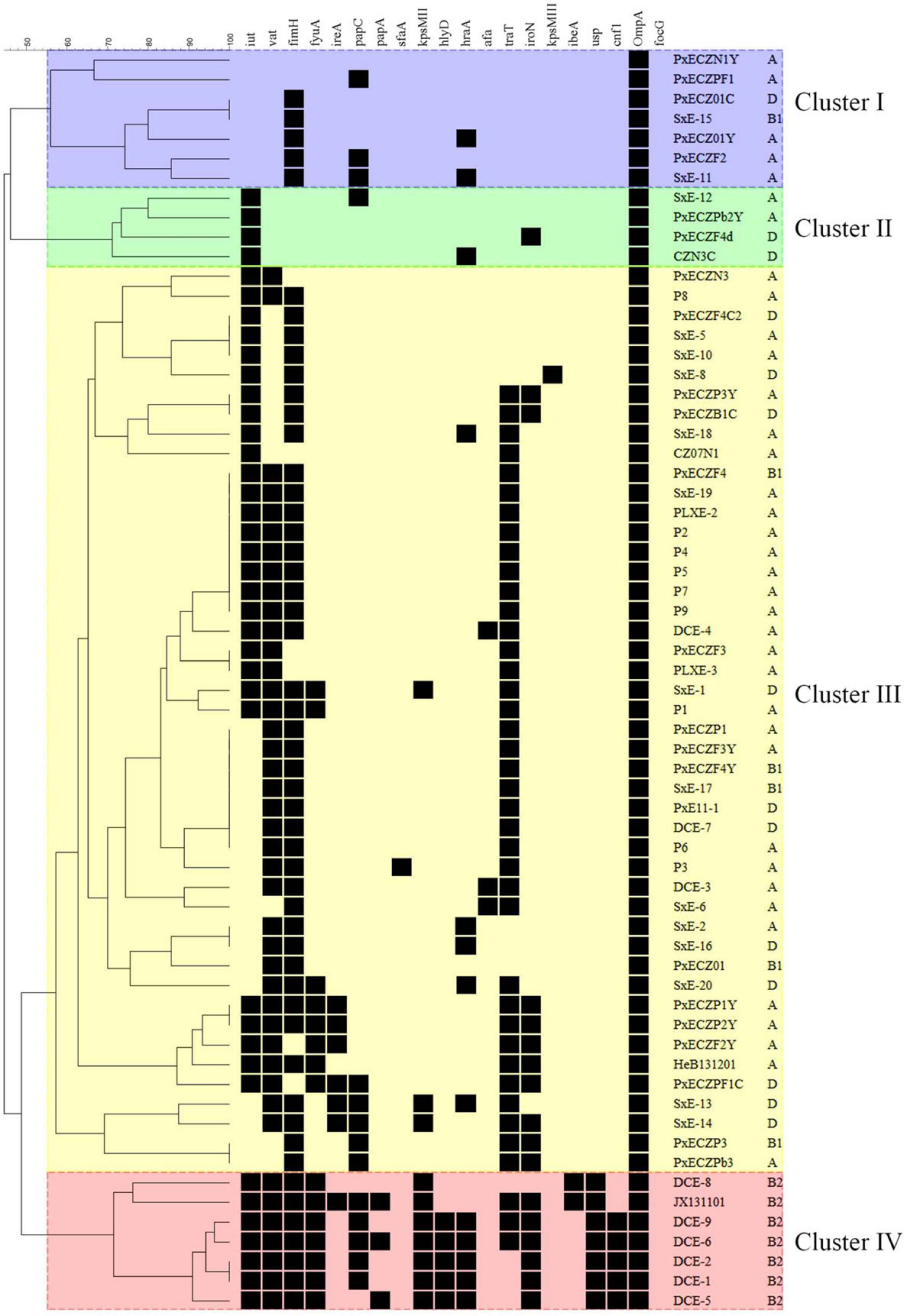
Clustering of the 64 ExPEC isolates based on the presence or absence of virulence-associated factors. Each column shows the results for a single gene. *Black*: gene present; *blank*: gene absent

Based on virulence gene profiles, four virulence clusters (I, II, III, IV) were identified using BioNumerics software according to a previous method (Fig. 1). Seven strains belonged to Cluster I, with an average of 2.43 virulence-associated genes per strain. Four stains belonged to Cluster II, with average of 2.7 virulence-associated genes. Cluster III contained 46 isolates, with an average of 4.92 virulence–associated genes. Cluster IV contained seven isolates with an average 12 virulence–associated genes. In general, the virulence of phylogenetic group B2 *E. coli* isolates is considered to be highest, followed by group D, which is in turn higher than that of phylogenetic groups A or B1 [12]. In this study, all phylogenetic group B2 isolates were found in Cluster IV. The dependency between Cluster IV and phylogenetic group B2 was significant (χ^2^ test, *P* < 0.05), but no significance was found between other clusters and phylogenetic groups. In addition, it was always observed the number of virulence-associated genes in an isolate was significantly correlated with its phylogenetic group, thus B2 isolates contained more virulence-associated genes than non-B2 isolates (χ^2^ test, *P* < 0.05). The prevalence of virulence-associated genes between clusters was also compared (Table 2), but no characteristic pattern was found for ExPEC. However, the presence of *hlyD, ibeA, usp, cnf1, papA* in Cluster IV is a difference from the other three clusters, indicating that those five genes are possible markers for the prediction of highly virulent ExPEC.

**Table 2:** Distribution of virulence factors classified according to clusters

Virulence factors	NO. of ExPEC isolates(%)				
	Total (*n* = 64;%)	Cluster I (*n* = 7;10.9%)	Cluster II (*n* = 4;6.25%)	Cluster III (*n* = 46;71.9% )	Cluster IV (*n* = 7;10.9%)
Siderophores					
*iutA*	39(60.1)	0(0)^a^	4(100)^b^	28(60.1)^b^	7(100)^b^
*iroN*	17(26.6)	0(0)^a^	1(25)^ab^	10(21.7)^a^	6(85.7)^b^
*fyuA*	15(23.4)	0(0)^a^	0(0)^a^	8(17.4)^a^	7(100)^b^
*ireA*	7(10.9)	0(0)	0(0)	6(13)	1(14.3)
Adhesins					
*fimH*	52(81.3)	5(71.4)^ac^	0(0)^b^	40(86.9)^c^	7(100)^c^
*papC*	14(21.9)	3(42.9)	1(25)	5(10.9)	5(71,4)
*papA*	3(4.7)	0(0)	0(0)	0(0)	3(42.9)
*sfaA*	1(1.6)	0(0)	0(0)	1(2.2)	0(0)
*hraA*	13(20.3)	2(28.6)	1(25)	5(10.9)	5(71.4)
*afa*	3(4.7)	0(0)	0(0)	3(6.5)	0(0)
Toxin					
*cnf1*	5(7.8)	0(0)^a^	0(0)^a^	0(0)^a^	5(71.4)^b^
*hlyD*	5(7.8)	0(0)^a^	0(0)^a^	0(0)^a^	5(71.4)^b^
*vat*	42(65.6)	0(0)^a^	0(0)^a^	35(76.1)^b^	7(100)^b^
Miscellaneous					
*ompA*	64(100)	7(100)	4(100)	46(100)	7(100)
*ups*	7(10.9)	0(0)^a^	0(0)^a^	0(0)^a^	7(100)^b^
*traT*	40(62.5)	0(0)^a^	0(0)^a^	37(80.4)^b^	3(42.9)^b^
Invasion					
*ibeA*	2(3.1)	0(0)	0(0)	0(0)	2(28.6)
Capsule					
*kpsMIII*	1(1.6)	0(0)	0(0)	1(2.2)	0(0)
*kpsMII*	10(15.7)	0(0)^a^	0(0)^a^	3(6.5)^a^	7(100)^b^

a,b,c *P* < 0.05

### MLST analysis

**Fig. 2:**
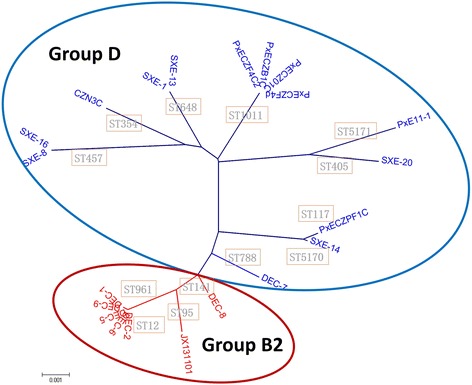
Phylogenetic organization of group B2 and D isolates of different multi locus sequence types based on the neighbor-joining method. Numbers at the nodes are bootstrap values and the *bar* represents 0.001 nucleotide changes. Group B2 strains gather together (*red circle*), as do group D strains (*blue circle*)

Among 21 isolates belonging to phylogenetic groups D and B2, 13 different STs were identified, including 11 known STs (ST1011, ST354, ST788, ST405, ST117, ST12, ST95, ST961, ST141, ST648 and ST457) (Additional file 1). Two novel STs were identified in this study, namely, ST5170 and ST5171. Four STs included all seven B2 group isolates, namely ST12, ST961, ST141, and ST95. Other STs were found for group D isolates. To further analyze the phylogenetic relationships of these 21 ExPEC isolates, a phylogenetic tree were generated (Fig. 2). The phylogenetic analysis showed that the group B2 and group D isolates could each be grouped (Fig. 2). All isolates in the group B2 section of the tree were classified into Cluster IV in the virulence genotyping analysis; the isolates in the group D part of the tree were from Clusters I, II and III (Fig. 2).

**Fig. 3:**
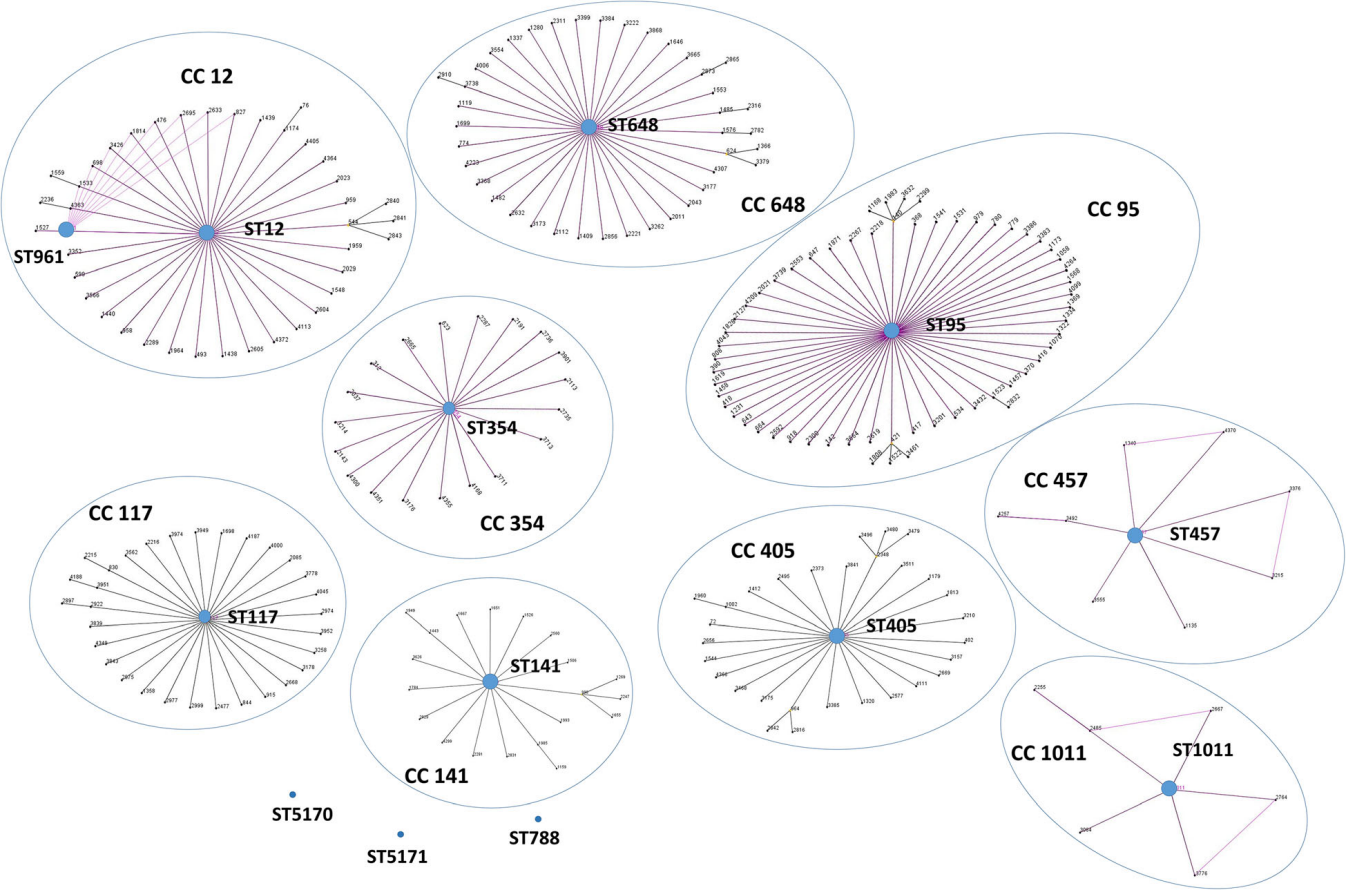
eBURST analysis of 13 STs of porcine extraintestinal *Escherichia coli*. The clonal structure of ExPEC is demonstrated by eBURST plots based on the profile of seven allelic housekeeping genes. Two STs differing in one locus but sharing the other six loci constitute a single locus variant (SLV); a double locus variant (DLV) means the STs contain two different loci. STs that did not include any SLVs with others are termed singletons. We observed nine main clonal complexes (CCs; CC12, CC648, CC354, CC141, CC405, CC457, CC1011, CC117 and CC95), and three singletons (ST788, ST5170 and ST5171). Strains from the same ancestor are grouped together in *blue circles*

eBURST analysis of these 21 isolates identified nine clonal complex (CCs), namely, CC1011, CC457, CC117, CC141, CC405, CC354, CC648, CC12 and CC95 (Fig. 3). ST774 is a single locus variant (SLV) of ST648, which is the primary founder of CC648. ST961 was connected to ST12 as a member of CC12. Three STs were not classified into any CCs, i.e., they were singletons (ST5170, ST788, and ST5171). Strains from CC12 and CC95 have been indicated to have strong pathogenicity and zoonotic potential (Table 3).

**Table 3:** Sequence types and clonal complex of group D and B2 isolates

Sequence type (ST)	Strains	Clusters	Clonal complex
ST1011	PxECZ01C	Cluster I	CC1011
ST1011	PxECZF4d	Cluster II	CC1011
ST354	CZN3C	Cluster II	CC354
ST1011	PxECZB1C	Cluster III	CC1011
ST1011	PxECZF4C2	Cluster III	CC1011
ST457	SXE-8	Cluster III	CC457
ST648 ST648	SXE-1	Cluster III	CC648
	SXE-13	Cluster III	CC648
ST5171	PxE11-1	Cluster III	None
ST788	DCE-7	Cluster III	None
ST457	SXE-16	Cluster III	CC457
ST405	SXE-20	Cluster III	CC405
ST117	PxECZPF1C	Cluster III	CC117
ST5170	SXE-14	Cluster III	None
ST961 ST961	DCE-9	Cluster IV	CC12
	DCE-1	Cluster IV	CC12
ST12	DCE-6	Cluster IV	CC12
ST12	DCE-2	Cluster IV	CC12
ST12	DCE-5	Cluster IV	CC12
ST141	DCE-8	Cluster IV	CC141
ST95	JX131101	Cluster IV	CC95

### Virulence evaluation of representative ExPEC isolates

Seven group B2 strains selected from Cluster IV showed high virulence in a mouse infection model (Table 4): the mortalities varied from 60% (3/5) to 100% (5/5) at a challenge dose of 4 × 10^7^ CFU; the mortalities were more diverse at a challenge dose of 4 × 10^6^ CFU, but of particular note, isolate JX131101 caused 100% mortality (5/5), indicating high virulence. The 14 group D strains showed obvious diversity in virulence: PxECZ01C and PxECZF4d did not cause any mortality at a dose of 4 × 10^7^ CFU, while strains SXE-20 and SXE-14 presented high virulence; all of group D strains have no pathogenicity at a challenge dose of 4 × 10^6^ CFU. No abnormality was found in mice in the control group injected with PBS.

**Table 4:** Experimental virulence of representative isolates in BALB/c mice

Clusters	Strains	Mortality		Phylogenetic groups	Virulence
		4 × 10^7^	4 × 10^6^		
Cluster I	PxECZ01C	0/5	0/5	D	None virulence
Cluster II	PxECZF4d	0/5	0/5	D	None virulence
Cluster II	CZN3C	2/5	0/5	D	Less virulence
Cluster III	PxECZF4C2	1/5	0/5	D	Less virulence
Cluster III	SXE-8	3/5	0/5	D	Less virulence
Cluster III	PxECZB1C	2/5	0/5	D	Less virulence
Cluster III	SXE-1	2/5	0/5	D	Less virulence
Cluster III	PxE11-1	2/5	0/5	D	Less virulence
Cluster III	DCE-7	1/5	0/5	D	Less virulence
Cluster III	SXE-16	3/5	0/5	D	Less virulence
Cluster III	SXE-20	4/5	0/5	D	Less virulence
Cluster III	PxECZPF1C	3/5	0/5	D	Less virulence
Cluster III	SXE-13	1/5	0/5	D	Less virulence
Cluster III	SXE-14	5/5	1/5	D	Virulence
Cluster IV	DCE-9	3/5	0/5	B2	Less virulence
Cluster IV	DCE-6	4/5	1/5	B2	Virulence
Cluster IV	DCE-2	3/5	0/5	B2	Less virulence
Cluster IV	DCE-5	4/5	3/5	B2	Virulence
Cluster IV	DCE-1	4/5	2/5	B2	Virulence
Cluster IV	DCE-8	4/5	2/5	B2	Virulence
Cluster IV	JX131101	5/5	5/5	B2	Virulence
	PBS	No mortality			

All these strains were able to kill BALB/c mice in doses of 10^8^ cfu, including PxECZ01C and PxECZF4d

## Discussion

The importance of ExPEC has been recognized, and many ExPEC related diseases in cows, humans and food have been reported in the North America and Europe. In China, ExPEC has become a frequent pathogen in the swine industry [13, 14]. However, understanding of the pathogenesis and epidemiology of porcine ExPEC is still limited. Some reports showed that >50% of ExPEC isolates from swine belong to phylogenetic groups A and B1 [15], which is consistent with our results. In this study, only a few isolates were classified as phylogenetic group B2, which is an obvious contrast with the fact that most ExPECs isolated from humans or companion animals belong to groups B2 and D [3]. In this study, the isolates were considered as ExPEC based organ origin. Johnson et al [16] proposed a molecular approach to delimit ExPEC, by considering the presence of 2 of the 5 virulence markers (*papA/papC, sfa/foc, afa/dra, iutA* and *kpsMTII*) in an *E. coli* strain as ExPEC. According to this criterion, 13 of 69 strains in this study were identified as ExPEC, which contained all 7 B2 strains (Additional file 3: Table S3). Notably, the prevalence of ExPEC that qualified to criterion was larger within high virulence strains (9/12, 75%; mortality ≥ 3) than within low virulence strains (2/9, 22.2%; mortality ≤ 2) in the mouse infection model. This indicated that Johnson’s criterion is more strict and selective compared to the robust standard we used in this study, and is able to identify strains with a higher potential to cause diseases.

A single virulence factor does not determine bacterial pathogenicity. Thus, in this study, 19 major virulence-associated genes of ExPEC were selected for a comprehensive analysis with phylogenetic investigation, aiming to reveal genotypic traits related to virulence. These 19 virulence-associated genes contribute to the fitness of ExPEC and increase its adaptability and competitiveness in colonizing host animals. Four clusters were identified in the genotyping analysis. Cluster IV included only B2 group isolates and showed a higher prevalence of virulence-associated genes than the other three clusters. Animal infection experiments confirmed higher virulence of isolates from Cluster IV, which is consistent with B2 group strains leading to serious diseases [17]. The distinction between B2 and non-B2 ExPEC is clear, shown by MLST, virulence genotyping and virulence assessment. In addition, group D ExPEC isolates are considered to be more pathogenic than groups A and B1, although in this study some group D strains presented similar virulence to group A or B1 isolates. Furthermore, group D isolates were mixed with group A and B1 isolates in Clusters I, II and III in the genotyping analysis. In MLST analysis, no obvious correlations were observed with genotypic cluster virulence in group D strains. Group D isolates thus present high diversity in genotype and phenotype.

MLST analysis can provide other information besides phylogenetic relationships [18]. After comparing our results with the MLST database (http://pubmlst.org/), we found STs in this study that have been reported to be present in diseases of birds, dogs and humans. For example, strain JX131101 belonged to ST95 in CC95; ST95 is the most important pathogenic sequence type in human-associated cases [19]. ST95 isolates are frequently reported to be related to newborn meningitis (NM) and uropathogenic infection (UTI) in Europe and the North America [20, 21]. Group D isolate CZN3C is assigned to ST354, which is a common ST type in isolates causing UTI and NM in humans, companion animals and swine [22, 23]. Some ST1011 isolates lead to UTI in avians and companion animals [24], but several group D ST1011 isolates showed low virulence in this study. Our data suggest that isolates from the same ST can have different pathogenic phenotypes, and indicate the diversity and complexity of pathogenic *E. coli*.

A study by Tan *et al.* showed that *fimH*, *traT* and *iutA* were highly prevalent in porcine ExPEC isolates, which is in consistent with our results, but the prevalence of *fyuA*, *cnf1*, *kpsMTII*, and *iroN* differ between our study and theirs; the *focG* gene was not detected in any isolates in this study, which is similar to Tan’s research [25]. Interestingly, the results of VR gene detection in ExPEC isolates from retail pork is quite different from that from pigs on farms [26]. This suggests that *E. coli* strains isolated from pork may originate from processes such as meat production or transportation.

We found five VR genes to be highly prevalent specifically in Cluster IV (which contained only group B2 isolates), namely *papA, hlyD, usp, ibeA* and *cnf1*, suggesting that these five genes may be closely related to strong pathogenicity. This result contains a risk factor that similar geographical of B2 isolated in this study might lead to a prevalence bias of virulence factors. Both *hlyD* and *cnf1* are well-known toxin genes in ExPEC. *ibeA* is associated with neonatal meningitis [27] and is an important virulence-associated factor widely distributed among APEC and NMEC [28]. *usp,* the uropathogenic-specific gene, was reported in a large number of strains from patients and animals with pyelonephritis or prostatitis [29]. Both our results and a previous study of virulence genotypes of canine ExPEC [11] indicate that *cnf1, hlyD, ups* and *kpsMII* could be possible marker genes for high virulence in ExPEC, although this point still needs to be verified in further study or using a larger collection of ExPEC isolates.

## Conclusions

In this study, a total of 19 VR genes in 64 Chinese porcine ExPEC strains were detected by PCR. Based on VR gene profiles, those isolates were divided into four clusters (named I to IV). Notably, all Cluster IV isolates were from group B2, and VR genes were remarkably more prevalent in group B2 than in groups A, B1 and D. The distinction between group B2 and non-B2 isolates in virulence and genotype accorded with MLST analysis. Considering Cluster IV isolates had the strongest pathogenicity in a mouse infection model, genes that were only found in group B2 strains (that *cnf1, hlyD, ups* and *kpsMII*) could be possible marker genes for high virulence in ExPEC. This study revealed the genetic differences between porcine ExPEC strains from China and enlarges the knowledge of the porcine ExPEC virulence.

## Methods

### Bacterial isolation and culture conditions

From April 2013 to August 2014, a total of 64 ExPEC strains were isolated from clinically diseased pigs in five provinces (Jiangsu, Zhejiang, Shandong, Hebei, Shanxi and Jiangxi) of China (Additional file 4: Table S2). All the isolates were recovered from extraintestinal organs, including spleen, lung, liver, kidney and brain, and MacConkey agar was used for initial purification. Isolates were grown in Luria-Bertani broth for 6 h and then stored in 50% glycerol at −70 °C until further characterization.

### Hemolytic activity tests

The hemolytic activities of clinical ExPEC isolates were determined by culture on 7% sheep blood agar. The formation of beta-hemolysin was observed.

### PCR detection of virulence-related genes and phylogenetic group identification

The genomic DNA of bacteria was extracted using an E.Z.N.A. bacterial DNA kit (Omega, Beijing, China) following the manufacturer’s instructions. Individual PCR assays were performed for 19 ExPEC VR genes, including *kpsMII, kpsMIII, iroN, fyuA, ireA, iutA, papC, hra, sfaS, papA, fimH, afa, hlyD, vat, cnf1, ompA, traT, usp* and *ibeA.* The phylogenetic group was determined by multiplex PCR with three pairs of primers, *chuA, yjaA* and *TspE4.C2* [30]. All PCR primers used in this study are listed in Table 5.

**Table 5:** Virulence genes primers for PCR in this study

PCR assay	Gene	Primer sequence	PCR product size/bp
Virulence- associated genes	*papA*	ATGGCAGTGGTGTCTTTTGGTG	717
		CGTCCCACCATACGTGCTCTTC	
	*papC*	GTGGCAGTATGAGTAATGACCGTTA	203
		ATATCCTTTCTGCAGGGATGCAATA	
	*iutA*	ATCGGCTGGACATCATGGGAAC	314
		CGCATTTACCGTCGGGAACGG	
	*vat*	CTTACCTCTCTGGCACTATCTG	1021
		GTCAGTGAACCGGCACC	
	*fimH*	TGCAGAACGGATAAGCCGTGG	508
		GCAGTCACCTGCCCTCCGGTA	
	*fyuA*	TGATTAACCCCGCGACGGGAA	787
		CGCAGTAGGCACGATGTTGTA	
	*ireA*	GATGACTCAGCCACGGGTAA	254
		CCAGGACTCACCTCACGAAT	
	*kpsMTII*	GCGCATTTGCTGATACTGTTG	272
		CATCCAGACGATAAGCATGAGCA	
	*hlyD*	CTCCGGTACGTGAAAAGGAC	904
		GCCCTGATTACTGAAGCCTG	
	*sfaS*	GTCTCTCACCGGATGCCAGAATAT	240
		GCATTACTTCCATCCCTGTCCTG	
	*hra*	GTAACTCACACTGCTGTCACCT	139
		CGAATCGTTGTCACGTTCAG	
	*cnf1*	CTTTACAATATTGACATGCTG	446
		TCGTTATAAAATCAAACAGTG	
	*afa*	TAAGGAAGTGAAGGAGCGTG	210
		CCGCCCTGAAGAAGTATCAC	
	*kpsMTIII*	TCCTCTTGCTACTATTCCCCCT	392
		AGGCGTATCCATCCCTCCTAAC	
	*ibeA*	AATGAGTGCCGCTCGTGAAGG	548
		CCCCCCAGTCTCCATATTTAG	
	*iroN*	CTCCGACGATGATAATGACGA	440
		TGGGACGTTTGGTAATGATGT	
	*ompA*	GATCAGTGCAGCACGCTGTTT	385
		GGGCGAAGCAGCTCCAGTAG	
	*traT*	CGATGAGCACAGCAATCAAGA	486
		CATTATCCGTTGTCACCGTTG	
	*usp*	GAGCGGTTATTTATTGAAATC	448
		CACAGCAGTCATCAACCACTG	

### Cluster analysis of virulence genotype

The presence or absence of putative virulence-associated genes among isolates was visualized using BioNumerics (version 6.6, Applied Maths, Kortrijk, Belgium) to study similarity among isolates, according to the method applied in a previous report [11]. These data were analyzed using a simple matching coefficient. Cluster analysis was performed by the unweighted pair group method using arithmetic averages (UPGMA).

### Multilocus sequence typing

MLST is a useful method to track genetic variation of microbes and survey epidemics. It provides an accurate and highly discriminatory genotyping system for bacteria. In this study, group D and group B2 ExPEC isolates were used to perform MLST tests. The DNA fragments of seven housekeeping genes (*purA, fumC, icd, mdh, recA, gyrB* and *adk*) were amplified by PCR and the products were sequenced. Allele sequence determination was performed using the *E. coli* MLST database (http://mlst.ucc.ie/mlst/dbs/E. coli). Any new alleles (i.e. those not recognized by the *E. coli* MLST system) were submitted to the database curator to be assigned new allele numbers. Sequence types (STs) were identified based on the allele profiles.

Phylogenetic analyses were conducted on the basis of the concatenation of seven MLST loci (*purA, fumC, icd, mdh, recA, gyrB* and *adk*) using the MEGA v4.1 software as previously described [31]. The result is presented as a neighbour-joining tree; the robustness of the groupings was assessed by bootstrap resampling of 1,000 replicates. eBURST (http://eburst.mlst.net) analysis was also performed to identify potential clonal complexes (CCs) and founders [18], and the overall population structures were determined.

### Statistical analysis

The prevalence of VR genes in ExPEC isolates in different clusters was compared using Fisher’s exact test (Statistica 5.0, StatSoft Inc., Tulsa, OK, USA). A chi-square test (χ^2^ test) was used to calculate the correlation between clusters, phylogenetic groups and the quantity of virulence-associated genes. Results were considered significant if *P* < 0.05.

### Animal infection experiments

*E. coli* strains belonging to groups D and B2 have a higher risk of causing severe disease. Using the results of genotype clustering, 21 group D and B2 ExPEC strains were tested in a mouse infection model. All isolates tested were grown in LB medium with shaking at 37 °C for about 4–5 h to reach a concentration of 10^9^ CFU/ml.

A total of 215 female BALB/c mice (5-weeks-old) were purchased from the Comparative Medicine Center of Yangzhou University. Mice were housed in the Animal Experimental Center of Nanjing Agricultural University, by a full-time staff. Temperature and humidity are controlled, food and water are accessible. The mice were divided into 43 groups, five mice per group. Mice in groups 1 to 42 were inoculated with the 21 selected strains by intraperitoneal injection (0.2 mL) at doses of 4 × 10^6^ or 4 × 10^7^ CFU per mouse. Group 43 was the uninoculated control and received phosphate buffered saline (PBS). The mice were monitored for survival twice a day for 1 week.

## Additional files

Additional file 1: MLST and VR genes profiles. (XLSX 16 kb)

Additional file 2: Table S1. Statistical analysis of associations between virulence-associated factores(VFs). *P* values by Fisher’s exact test, shown only where < .10. *P* < .10 reflects statistical significance ;*P* values between .01 and .05 reflect possible statistical significance. (DOCX 19 kb)

Additional file 3: Table S2. The isolation sources of strains in this study. (DOCX 18 kb)

Additional file 4: Table S3. The information of isolates identified as ExPEC with Johnson et al’s criterion (DOCX 15 kb)

## Abbreviations

ExPEC: Extraintestinal pathogenic *Escherichia coli*
MLST: Multilocus sequence typing
VR: Virulence-related
