# Supplementary material for: Characterization and virulence clustering analysis of extraintestinal pathogenic Escherichia coli isolated from swine in China
Source: BMC Vet Res. 2017 Apr 8;13:94. doi: 10.1186/s12917-017-0975-x (PMC5385051; doi:10.1186/s12917-017-0975-x)
Supplement: Supplementary file 2 — Statistical analysis of associations between virulence-associated factores(VFs). P values by Fisher’s exact test, shown only where < .10. P < .10 reflects statistical significance ;P values between .01 and .05 reflect possible statistical significance. (DOCX 19 kb) [file 12917_2017_975_MOESM2_ESM.docx]

**Table S1. Statistical analysis of associations between virulence-associated factores(VFs).** P values by Fisher’s exact test, shown only where <.10. P<.10 reflects statistical significance ;P values between .01 and .05 reflect possible statistical significance.

|  | iutA | vat | fimH | fyuA | ireA | papC | papA | sfaA | kpsMII | hlyD | hraA | afa | traT | iroN | kpsMIII | ibeA | ups | cnf1 | ompA |
| --- | --- | --- | --- | --- | --- | --- | --- | --- | --- | --- | --- | --- | --- | --- | --- | --- | --- | --- | --- |
| iutA | NA |  |  |  |  |  |  |  |  |  |  |  |  |  |  |  |  |  |  |
| vat | 0.125 | NA |  |  |  |  |  |  |  |  |  |  |  |  |  |  |  |  |  |
| fimH | 0.006 | 0.022 | NA |  |  |  |  |  |  |  |  |  |  |  |  |  |  |  |  |
| fyuA | <0.001 | 0 | 0 | NA |  |  |  |  |  |  |  |  |  |  |  |  |  |  |  |
| ireA | 0 | 0 | 0 | 0.033 | NA |  |  |  |  |  |  |  |  |  |  |  |  |  |  |
| papC | <0.001 | 0 | 0 | 0.163 | 0.048 | NA |  |  |  |  |  |  |  |  |  |  |  |  |  |
| papA | 0 | 0 | 0 | 0.002 | 0.114 | 0.003 | NA |  |  |  |  |  |  |  |  |  |  |  |  |
| sfaA | 0 | 0 | 0 | <0.001 | 0.028 | <0.001 | 0.249 | NA |  |  |  |  |  |  |  |  |  |  |  |
| kpsMII | 0 | 0 | 0 | 0.096 | 0.153 | 0.12 | 0.029 | 0.004 | NA |  |  |  |  |  |  |  |  |  |  |
| hlyD | 0 | 0 | 0 | 0.011 | 0.23 | 0.017 | 0.222 | 0.089 | 0.087 | NA |  |  |  |  |  |  |  |  |  |
| hraA | 0 | 0 | 0 | 0.155 | 0.068 | 0.167 | 0.006 | <0.001 | 0.144 | 0.026 | NA |  |  |  |  |  |  |  |  |
| afa | 0 | 0 | 0 | 0.002 | 0.114 | 0.003 | 0.32 | 0.249 | 0.029 | 0.222 | 0.006 | NA |  |  |  |  |  |  |  |
| traT | 0.142 | 0.137 | 0.009 | <0.001 | 0 | 0 | 0 | 0 | 0 | 0 | 0 | 0 | NA |  |  |  |  |  |  |
| iroN | <0.001 | <0.001 | 0 | 0.149 | 0.014 | 0.135 | <0.001 | <0.001 | 0.056 | 0.004 | 0.118 | <0.001 | <0.001 | NA |  |  |  |  |  |
| kpsMIII | 0 | 0 | 0 | <0.001 | 0.023 | <0.001 | 0.249 | 0.504 | 0.004 | 0.089 | <0.001 | 0.249 | 0 | <0.001 | NA |  |  |  |  |
| ibeA | 0 | 0 | 0 | <0.001 | 0.066 | <0.001 | 0.317 | 0.378 | 0.013 | 0.163 | 0.002 | 0.317 | 0 | <0.001 | 0.378 | NA |  |  |  |
| ups | 0 | 0 | 0 | 0.033 | 0.222 | 0.048 | 0.114 | 0.029 | 0.153 | 0.199 | 0.068 | 0.114 | 0 | 0.014 | 0.028 | 0.066 | NA |  |  |
| cnf1 | 0 | 0 | 0 | 0.011 | 0.199 | 0.0167 | 0.222 | 0.089 | 0.087 | 0.256 | 0.026 | 0.222 | 0 | 0.004 | 0.089 | 0.163 | 0.199 | NA |  |
| ompA | 0 | 0 | <0.001 | 0 | 0 | 0 | 0 | 0 | 0 | 0 | 0 | 0 | 0 | 0 | 0 | 0 | 0 | 0 | NA |
