# Supplementary material for: Characterization and virulence clustering analysis of extraintestinal pathogenic Escherichia coli isolated from swine in China
Source: BMC Vet Res. 2017 Apr 8;13:94. doi: 10.1186/s12917-017-0975-x (PMC5385051; doi:10.1186/s12917-017-0975-x)
Supplement: Supplementary file 3 — The isolation sources of strains in this study. (DOCX 18 kb) [file 12917_2017_975_MOESM3_ESM.docx]

| **Strains** | **Isolation source (province)** | **Strains** | **Isolation source (province)** |
| --- | --- | --- | --- |
| PxECZ01C | Anhui | PxECZB1C | Shandong |
| PxECZ01Y | Anhui | PxECZP3Y | Shandong |
| P1 | Anhui | PxECZB1C | Shandong |
| P2 | Anhui | PxECZF4 | Shandong |
| P3 | Anhui | PxECZF3 | Shandong |
| P4 | Anhui | PxECZP1 | Shandong |
| P5 | Anhui | PxECZF3Y | Shandong |
| P6 | Anhui | PxECZF4Y | Shandong |
| P7 | Anhui | PxECZP1Y | Shandong |
| P8 | Anhui | PxECZP2Y | Shandong |
| P9 | Anhui | PxECZF2Y | Shandong |
| PxECZ01 | Anhui | PxECZPF1C | Shandong |
| HeB131201 | Hebei | PxECZP3 | Shandong |
| CZ07N1 | Jiangsu | PxECZPb3 | Shandong |
| CZN3C | Jiangsu | PLXE-2 | Not recorded |
| Dec-1 | Jiangsu | PLXE-3 | Not recorded |
| Dec-2 | Jiangsu | PxE11-1 | Not recorded |
| Dec-3 | Jiangsu | SxE-15 | Zhejiang |
| Dec-4 | Jiangsu | SxE-11 | Zhejiang |
| Dec-5 | Jiangsu | SxE-12 | Zhejiang |
| Dec-6 | Jiangsu | SxE-5 | Zhejiang |
| Dec-7 | Jiangsu | SxE-10 | Zhejiang |
| Dec-8 | Jiangsu | SxE-8 | Zhejiang |
| Dec-9 | Jiangsu | SxE-18 | Zhejiang |
| JX131101 | Jiangxi | SxE-19 | Zhejiang |
| PxECZN1Y | Shandong | SxE-1 | Zhejiang |
| PxECZPF1 | Shandong | SxE-17 | Zhejiang |
| PxECZF2 | Shandong | SxE-6 | Zhejiang |
| PxECZPb2Y | Shandong | SxE-2 | Zhejiang |
| PxECZF4d | Shandong | SxE-16 | Zhejiang |
| PxECZN3 | Shandong | SxE-20 | Zhejiang |
| PxECZF4C2 | Shandong | SxE-13 | Zhejiang |
| PxECZP3Y | Shandong | SxE-14 | Zhejiang |

**Table S2. The isolation sources of strains in this study**
