# Supplementary material for: Characterization and virulence clustering analysis of extraintestinal pathogenic Escherichia coli isolated from swine in China
Source: BMC Vet Res. 2017 Apr 8;13:94. doi: 10.1186/s12917-017-0975-x (PMC5385051; doi:10.1186/s12917-017-0975-x)
Supplement: Supplementary file 4 — The information of isolates identified as ExPEC with Johnson et al’s criterion (DOCX 15 kb) [file 12917_2017_975_MOESM4_ESM.docx]

| Strains | Phylogenetic group | STs | Isolation source (province) | Mortality rate | | Virulence genes | | | | |
| --- | --- | --- | --- | --- | --- | --- | --- | --- | --- | --- |
|  |  |  |  | 4 × 10^7^ | 4 × 10^6^ | *papA/C* | *sfa/foc* | *afa/dra* | *iutA* | *kpsM II* |
| DCE-4 | A | Unknown | Jiangsu | Unknow | Unknow |  |  | + | + |  |
| SXE-12 | A | Unknown | Zhejiang | Unknow | Unknow | + |  |  | + |  |
| SXE-1 | D | ST648 | Zhejiang | 2/5 | 0/5 |  |  |  | + | + |
| SXE-13 | D | ST648 | Zhejiang | 1/5 | 0/5 | + |  |  |  | + |
| SXE-1 | D | ST5170 | Zhejiang | 5/5 | 0/5 | + |  |  |  | + |
| PxECZPF1C | D | ST117 | Shandong | 3/5 | 0/5 | + |  |  | + |  |
| DCE-1 | B2 | ST961 | Jiangsu | 4/5 | 1/5 | + |  |  | + | + |
| DCE-2 | B2 | ST12 | Jiangsu | 3/5 | 0/5 | + |  |  | + | + |
| DCE-5 | B2 | ST12 | Jiangsu | 4/5 | 3/5 | + |  |  | + | + |
| DCE-6 | B2 | ST12 | Jiangsu | 4/5 | 1/5 | + |  |  | + | + |
| DCE-8 | B2 | ST141 | Jiangsu | 4/5 | 2/5 |  |  |  | + | + |
| DCE-9 | B2 | ST961 | Jiangsu | 3/5 | 0/5 | + |  |  | + | + |
| JX131101 | B2 | ST95 | Jiangxi | 5/5 | 5/5 | + |  |  | + | + |

**Table S3. The information of isolates identified as ExPEC with Johnson et al’s criterion**
